# Case report and literature analysis: Autoimmune cerebellar ataxia associated with homer-3 antibodies

**DOI:** 10.3389/fneur.2022.951659

**Published:** 2022-07-26

**Authors:** Qisi Wu, Beibei Gong, Anan Jiang, Xinyue Qin

**Affiliations:** ^1^Department of Neurology, The First Affiliated Hospital of Chongqing Medical University, Chongqing, China; ^2^Department of Radiology, The First Affiliated Hospital of Chongqing Medical University, Chongqing, China

**Keywords:** autoimmune cerebellar ataxia, Homer-3 antibody, brain MRI enhanced abnormalities, relapse, rituximab

## Abstract

**Objective:**

We present a case of autoimmune cerebellar ataxia (ACA) associated with Homer protein homolog 3 (Homer-3) antibodies. Then, a review of the literature was conducted to summarize its clinical spectrum to improve clinicians' understanding of this rare entity.

**Case presentation:**

A 25-year-old man suffered from the subacute onset of cerebellar ataxia and psychiatric symptoms with abnormalities in the cerebellum on initial brain MRI and Homer-3 antibodies titers of 1:100 in the serum. His neurological symptoms did not improve after intravenous methylprednisolone but significantly improved following plasma exchange with a modified Rankin Scale (mRS) score of 1. However, 5 months later, he experienced relapse during oral prednisone tapering with enhanced cerebellar lesions and obvious cerebellar atrophy on repeated MRI. Various immunomodulatory approaches, including corticosteroids and plasma exchange, were utilized with no improvement. Then rituximab was given for the first time to treat Homer-3 autoimmunity with partial improvement of symptoms. However, the patient remained profoundly disabled with an mRS score of 4.

**Conclusion:**

ACA associated with Homer-3 antibodies may have a suboptimal response to corticosteroid therapy. More intense immunotherapy such as rituximab may contribute to the improvement of cerebellar syndrome. Relapsing courses and presentation of cerebellar atrophy may suggest a poor prognosis in this entity.

## Introduction

Autoimmune cerebellar ataxia (ACA) associated with Homer protein homolog 3 (Homer-3) antibodies is a rare disease. To the best of our knowledge, only 11 cases have been reported in the literature (1–6). The whole clinical spectrum and potential treatment options remain obscure. Here, we present a well-characterized case of Homer-3 autoimmunity, a 25-year-old man who experienced clinical relapse with enhanced abnormalities in the cerebellum on brain magnetic resonance imaging (MRI) that has not been reported in previous studies, and in whom rituximab was initiated for the first time with partial improvement of symptoms. Then, we reviewed and analyzed all ACA cases associated with Homer-3 antibodies, summarizing the clinical presentations, diagnostic considerations, imaging findings, treatment, and prognosis of this disease. Our purpose was to aid in the clinical understanding of this rare entity.

## Case presentation

**Figure 1:**
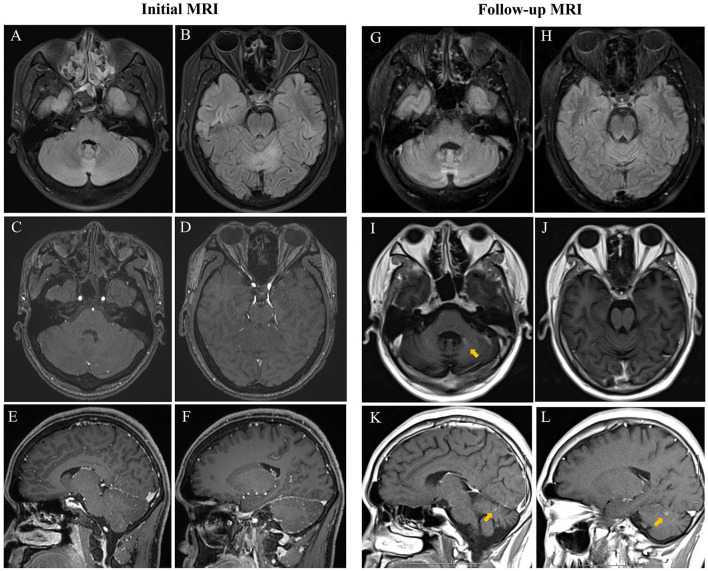
Initial cerebral MRI showed increased signal in the vermis and both bilateral cerebellar hemispheres on FLAIR **(A,B)** without enhancement on contrast-enhanced T1-weighted sequence **(C–F)**. Repeated MRI showed an increase of FLAIR hyperintensity of cerebellar lesions **(G)** and progression in size of the vermis lesions **(H)** with slightly enhanced T1-weighted signals on both axial and sagittal view and obvious cerebellar atrophy **(I–L)**.

**Figure 2:**
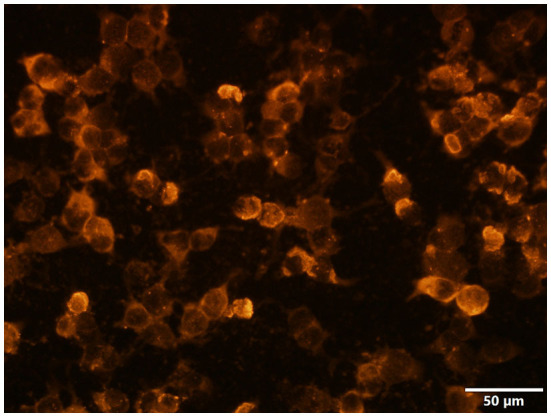
The Homer-3 antibody of serum was positive: the red marker represented Homer-3 antibodies (CBA, indirect immunofluorescence) (×400). The scale bar was 50 μm.

A 25-year-old man was admitted because of the subacute onset of vertigo, nausea, and vomiting for 2 weeks with slurred speech and unsteady gait for 1 week. He denied symptoms of previous infectious diseases. His past medical and family history was unremarkable. In the clinic, the patient was alert but unable to walk without help. Neurological examination demonstrated dysarthria, bilateral horizontal nystagmus, moderate limb dysmetria, and gait ataxia with a scale for the assessment and rating of ataxia (SARA) score of 20. The modified Rankin Scale (mRS) score on admission was 4. Initial brain MRI was performed and showed hyperintensities in the vermis and bilateral cerebellar hemispheres on fluid-attenuated inversion recovery (FLAIR) without enhancement (Figures 1A–F). Cerebrospinal fluid (CSF) study revealed an elevated opening pressure of 200 mmH_2_O, mild pleocytosis (white blood cell count 50/ul, 88% lymphocytes; reference range: 0–8/ul), elevated protein level (0.63 g/L; reference range: 0.1–0.45 g/L) and increased CSF IgG index (74.7 mg/L; reference range: 10–40 mg/L). Oligoclonal bands (OCB) were negative. Peripheral blood cell counts, electrolytes, liver and kidney functions, and levels of lactate, ammonia, and vitamins B1 and B12 were all normal. Screening for viral and bacterial infections, tumors, and thyroid diseases was negative. The further laboratory workup of autoantibodies was notable for positive serum Homer-3 antibodies with a titer of 1:100, whereas the CSF sample was negative by using the indirect immunofluorescence technique (IIFT) employing transfected HEK293 cells (Figure 2). Serum and CSF paraneoplastic antibodies (anti-Hu, Yo, Ri, Ma1/2, CV2, Tr, SOX1, Zic4, and amphiphysin), autoimmune encephalitis antibodies (anti-NMDAR, AMPAR, LGI1, GABA_B_, GAD65, CASPR2, IgLON5, DPPX, GlyR1, DRD2, and mGluR5) and serological markers specific for collagen diseases (antinuclear, anti-DNA, anti-Sm, anti-RNP, anti-SSA, anti-SSB, antineutrophil cytoplasmic antibodies) were either negative or in the normal range. Diagnosis of autoimmune cerebellar ataxia was made, and he was given intravenous methylprednisolone 1,000 mg for 3 days, followed by 500 mg for 3 days, and taped to oral prednisone 70 mg per day. However, no clinical improvement but a dysarthria deterioration was observed. His slurred speech could hardly be understood then with a score of 22 on SARA and a score of 4 on mRS. Moreover, he further developed psychiatric symptoms including difficulties with emotional control and impaired communication. Given these reasons, three circles of plasma exchanges (2,000 ml each time) were carried out at intervals of 2 to 3 days with significant improvement of psychiatric symptoms and slurred speech. And the patient could gradually walk alone. He was discharged with oral prednisone on a chronic basis and followed up at a local hospital. A score of 14.5 on SARA and a score of 1 on mRS were registered then.

**Figure 3:**
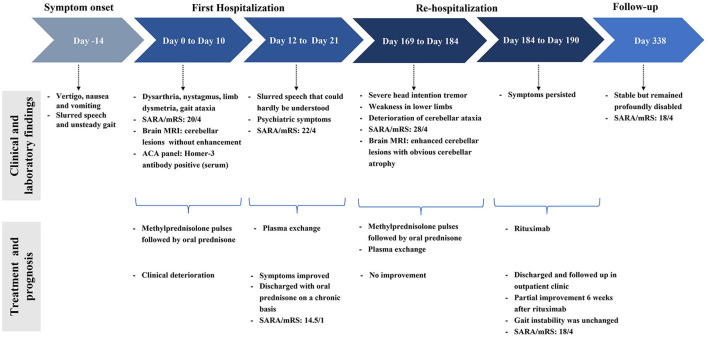
Case report timeline. CBA, cell-based assay; FLAIR, fluid-attenuated inversion recovery; MRI, magnetic resonance imaging; mRS, modified Rankin Scale; SARA, scale for the assessment and rating of ataxia.

However, 5 months later, the patient was admitted to our hospital again as he had severe head intention tremors and weakness in lower limbs for a month while he was currently on a daily dose of 30 mg of prednisone. He also complained of deterioration of slurred speech and gait instability. On neurological examination, he could barely stand with a SARA score of 28 and an mRS score of 4. CSF analysis revealed an increased protein level of 0.96 g/L. Repeated screening for neoplasia has been negative. Remarkably, repeated cerebral MRI indicated obvious cerebellar atrophy and worsened cerebellar lesions with gadolinium enhancement (Figures 1G–L). He received high-dose intravenous corticosteroids and cycles of plasma exchange with no response. Thus, an intravenous infusion of rituximab was subsequently administered. Progressive clinical improvement was noticed with near resolution of head intention tremor and weakness of lower limbs 6 weeks after rituximab. However, the gait instability was unchanged. After 5 months of follow-up, the patient was stable but remained profoundly disabled with an mRS score of 4 and a SARA score of 18 as he still required help when walking. The case report timeline is presented in Figure 3.

## Discussion

ACA is an important cause of acquired cerebellar ataxia both in children and adults (7). Recent studies have identified several relevant antibodies serving as biomarkers of ACA, which were comprised of paraneoplastic antibodies such as anti-Hu, anti-Yo, Anti-Ri, amphiphysin, and other antibodies, and non-paraneoplastic antibodies including GAD65, AP3B2, neurochondrin, septin-5 and Homer-3 antibodies (2, 8–11). Homer-3 is expressed at a high level on Purkinje cell dendrites spines. It is the scaffold protein interacting with metabotropic glutamate receptor 1 (mGluR1) and intracellular calcium channel (ITPR1), thereby controlling the ability of the mGLuR1 receptor to trigger calcium responses (12). As a protein involved in calcium-related glutamate signaling pathways, Homer-3 has been recently identified as a new antigenic target of the immune response against Purkinje cells and causes cerebellar ataxia (1, 9). However, the direct effects of Homer-3 antibodies on cultured neurons have not yet been examined. Whether Homer-3 antibodies are directly attributed to the immunopathogenesis of Homer-3-associated autoimmunity or their pathogenicity is mediated by T lymphocytes is unclear so far (8).

### Clinical presentations

A total of twelve cases, including ours, have been described in this study (Table 1) (1–6). Patients' age ranged from 10 to 84 years (mean ± SD: 43.92 ± 23.01), seven (58.33%) were women. Then, two patients had a history of prodromal infection. The onset was subacute/acute in nine and insidious in three patients. Cerebellar symptoms were noted in all patients, including vertigo, nausea, vomiting, nystagmus, head intention tremor, speech dysarthria, limb dysmetria, and gait ataxia. A total of five patients exhibited symptoms of encephalopathy including psychosis (*n* = 3), seizures (*n* = 2), confusion (*n* = 1) and cognitive impairment (*n* = 2). Other extracerebellar features included myeloradiculopathy (*n* = 2), REM sleep behavior disorder (RBD) (*n* = 2) and autonomic dysfunction (*n* = 2). Except for one patient with pulmonary nodules of potential malignancy, extensive studies failed to reveal any tumor in these patients.

**Table 1:** Review all reported ACA cases with Homer-3 antibodies.

Case	Age/Gender	Onsetc	Neurological symptoms	Tumor	Initial/Follow-up MRI (months from onset)	Detection of Homer-3 antibodies	CSF WBC (/ul)/protein(g/l)/ intrathecal IgG synthesis	Treatment	Outcome/mRS (months from onset)
Case 1	25/M	Subacute	Cerebellar syndrome and psychiatric symptoms	No	FLAIR hyperintensities in cerebellar hemispheres and vermis/worsened cerebellar lesions with enhancement and obvious cerebellar atrophy (5)	Serum	50/0.63/increased IgG index	CS, PLEX, rituximab	Partially improved but relapsed/4(11)
**Zuliani et al.** (1)									
Case 2	65/F	Subacute	Cerebellar syndrome	No	Normal/NA	Serum*	27/NA/increased IgG index	CS	No improvement/NA(68)
**Höftberger et al.** (2)									
Case 3	38/M	Acute	Cerebellar syndrome and complex partial seizures	No	Normal/mild cerebellar atrophy (10)	Serum*	60/1.11/no	IVIg, CS	Partially improved/2(24)
**Xu et al.** (3)									
Case 4	51/F	Insidious	Cerebellar syndrome	No	Cerebellar atrophy/cerebellar atrophy (48)	Serum*	0/0.41/OCB positive	CS, MMF	Partially improved/3(12)
**Liu et al.** (4)									
Case 5	46/F	Insidious	Cerebellar syndrome	No	Cerebellar atrophy /worsened cerebellar atrophy (98)	Serum and CSF	0/0.41/OCB positive	CS, MMF	Partially improved/5(98)
Case 6	50/F	Subacute	Cerebellar syndrome and RBD	No	Normal/Cerebellar and pontine atrophy (16)	Serum	2/0.3/no	CS, MMF	Partially improved/2(31)
Case 7	14/M	Subacute	Cerebellar syndrome, cognitive impairment and myeloradiculopathy	No	Diffuse T2 hyperintensity in bilateral cerebral hemispheres /decrease of T2 hyperintensity (8)	Serum	21/0.61/OCB positive	IVIg, CS	Partially improved but relapsed twice/3(40)
Case 8	65/M	Insidious	Cerebellar syndrome and RBD	No	Cerebellar and pontine atrophy/worsened cerebellar and pontine atrophy (24)	Serum	30/1.136/NA	IVIg, CS, PLEX	Deteriorated/4(64)
Case 9	84/F	Subacute	Cerebellar syndrome	Potential malignant pulmonary nodules	Normal/normal (9)	Serum	6/0.48/NA	CS	No improvement/2(23)
Case 10	59/F	Subacute	Cerebellar syndrome, psychosis, seizure, confusion and radiculoneuropathy	No	FLAIR hyperintensity in bilateral cerebral cortex/normal (10)	Serum	2/0.17/no	IVIg, CS	Improvement followed by relapse/4(11)
**Miao et al.** (5)									
Case 11	20/F	Subacute	Cerebellar syndrome	No	T2 hyperintensities in the right cerebellar hemisphere/normal (1.5)	Serum and CSF	139/1.67/OCB positive	IVIg, CS, MMF	Obviously improved/3(2)
**Kuang et al.** (6)									
Case 12	10/M	Subacute	Cerebellar syndrome, cognitive impairment and irritability	NA	T2 and FLAIR hyperintensities in cerebellar hemispheres and vermis/NA	CSF	30/0.3/NA	IVIg, CS	Obviously improved/1(NA)

* The antibody panel was not performed in CSF.

ACA, autoimmune cerebellar ataxia; CS, corticosteroid; FLAIR, fluid attenuated inversion recovery; IVIg, IV immunoglobulin; MMF, mycophenolate mofetil; MRI, magnetic resonance imaging; mRS, modified Rankin Scale; NA, not available; OCB, oligoclonal bands; PLEX, plasma exchange; WBC, white blood cell.

### Diagnostic investigations

The detailed CSF data were available for all 12 patients with inflammatory changes: lymphocytic pleocytosis was noted in seven patients (cell counts 21–139/ul), elevated protein was noted in four patients (0.61–1.67 g/l), and intrathecal IgG synthesis was elevated in six patients. Besides, Homer-3 antibodies were detected in CSF of three patients (the CSF antibody panel was not performed in case 2, case 3, and case 4) and in serum of 11 patients, which indicated that serum and CSF testing is mandatory when ACA is considered. Ideally, both cell-based and tissue-based assays should be used to test for Homer-3 antibodies. However, we did not conduct a tissue-based assay, which was a limitation of this report.

Initial brain MRI was performed in all patients with variable manifestations, which were normal (*n* = 4), and showed bilateral cerebral/cerebellar abnormalities (*n* = 5) or cerebellar atrophy (*n* = 3). Repeated MRI was obtained in 10 patients on follow-up at 1.5–98 months. The cerebral/cerebellar lesions were reported to shrink after treatment in three patients. Nevertheless, the follow-up MRI in our patient showed enhanced cerebellar lesions which have not been reported in previous studies, indicating evidence of cerebellar inflammation. Moreover, it is important to note that in more than half of the patients (6 out of 10), the repeated MRI disclosed cerebellar or pontine atrophy after comprehensive immunotherapy, which is probably the result of the secondary degeneration of cerebellar circuits after cerebellar inflammation (4, 13, 14).

### Treatment and prognosis

There are no standards for the treatment of Homer-3 autoimmunity. First-line immunotherapies in the acute phase including corticosteroids, intravenous immunoglobulins (IVIg), and plasma exchange may be beneficial (1–6). Besides, long-term immunosuppression such as oral prednisone and mycophenolate mofetil (MMF) was administered in some patients for the possibility of long-term clinical benefit, which was reported to halt and minimize cerebellar ataxia in Homer-3 autoimmunity (2–5). However, the response to immunotherapy was equivocal. In our patient, treatment with methylprednisolone did not improve the symptoms in the acute phase and maintenance therapy of oral prednisone did not prevent the occurrence of clinical relapse and cerebellar atrophy. In this situation, intravenous rituximab was given for the first time to treat Homer-3 autoimmunity, and partial improvement was observed. This may indicate that more intense immunotherapy such as rituximab could be a second choice when the first-line treatment did not work out.

The overall outcome of this disease was poor. Only 4 patients were reported to achieve a good functional outcome (mRS ≤2) and almost all the patients ended up with neurological sequelae. This is partially explained by cerebellar atrophy, a possible complication of ACA associated with Homer-3 antibodies (1–6, 14). Moreover, it is important to highlight that three patients including our patient experienced clinical relapse during corticosteroid tapering or weaning or after they stopped IVIg infusion (4). The fact that these patients remained profoundly disabled may imply that relapsing courses can lead to a poor prognosis.

## Conclusion

In patients with Homer-3 autoimmunity, extensive studies failed to reveal any tumor, MRI findings were variable, CSF always presented with inflammatory changes, Homer-3 antibodies were detected in serum or CSF and the response to immunotherapy treatment was equivocal. Intravenous rituximab may partially improve cerebellar symptoms, especially in relapsing cases. The neurologic prognosis depends on multiple factors. Relapsing courses and presentation of cerebellar atrophy may suggest that recovery will be incomplete.

## Data availability statement

The original contributions presented in the study are included in the article/supplementary material, further inquiries can be directed to the corresponding author/s.

## Ethics statement

The studies involving human participants were reviewed and approved by Ethics Committee of the First Affiliated Hospital of Chongqing Medical University. The patients/participants provided their written informed consent to participate in this study. Written informed consent was obtained from the individual(s) for the publication of any potentially identifiable images or data included in this article.

## Author contributions

QW made substantial contributions to study concept and design, interpretation of clinical data, drafting of the manuscript, and fund obtaining. BG made contributions to the acquisition, analysis, and interpretation of imaging data. AJ conducted the literature review and drafted the manuscript. QW and XQ were involved in revising the manuscript critically and have given final approval for the version to be published. All authors read and approved the manuscript.

## Funding

This work was supported by the Natural Science Foundation of Chongqing (grant no. cstc2020jcyj-msxmX0765).

## Conflict of interest

The authors declare that the research was conducted in the absence of any commercial or financial relationships that could be construed as a potential conflict of interest.

## Publisher's note

All claims expressed in this article are solely those of the authors and do not necessarily represent those of their affiliated organizations, or those of the publisher, the editors and the reviewers. Any product that may be evaluated in this article, or claim that may be made by its manufacturer, is not guaranteed or endorsed by the publisher.
