# Corrigendum: Case report and literature analysis: Autoimmune cerebellar ataxia associated with homer-3 antibodies

**DOI:** 10.3389/fneur.2023.1187907

**Published:** 2023-03-28

**Authors:** Qisi Wu, Beibei Gong, Anan Jiang, Xinyue Qin

**Affiliations:** ^1^Department of Neurology, The First Affiliated Hospital of Chongqing Medical University, Chongqing, China; ^2^Department of Radiology, The First Affiliated Hospital of Chongqing Medical University, Chongqing, China

**Keywords:** autoimmune cerebellar ataxia, Homer-3 antibody, brain MRI enhanced abnormalities, relapse, rituximab

In the published article, there was an error in the Funding statement. The grant number was incorrect. The correct Funding statement appears below.

